# The rectification of an electrical synapse can change the functional output of a pattern-generating circuit

**DOI:** 10.1186/1471-2202-14-S1-P259

**Published:** 2013-07-08

**Authors:** Gabrielle J Gutierrez, Eve Marder

**Affiliations:** 1 Volen Center for Complex Systems, Brandeis University, Waltham, MA 02454, USA

## 

Electrical synapses are known to promote synchrony and speed in neuronal systems. However, other studies have found that electrical synapses can have the opposite effect by desynchronizing network neurons. Although they are mechanistically simple, electrical synapses can have unintuitive effects on neuronal network activity. The present study aims to further our understanding of electrical synapses by exploring their rectification in a neuronal circuit model. Rectification is the asymmetrical passage of current through a gap junction between two neurons and it is often likened to a diode in an electrical circuit. Rectifying electrical synapses have been shown to support specialized functions in several biological preparations [1]. We investigate how rectification affects the functional output of a 5-cell, pattern-generating, model network and its sensitivity to synaptic modulation. The circuit is composed of heterogeneous neurons with different intrinsic oscillation frequencies. Neurons were modeled as Morris-Lecar [2] neurons modified by a hyperpolarization-activated current as in a previous study [3].

**Figure 1:**
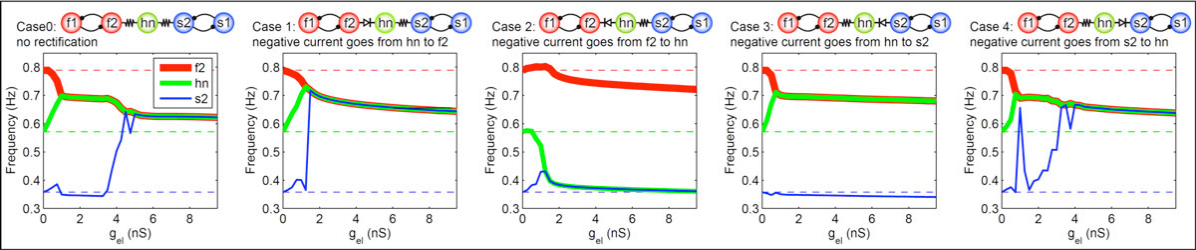
**In a 5-cell pattern-generating circuit of heterogeneous neurons, f1 and f2 are intrinsically fast oscillators, s1 and s2 are intrinsically slow oscillators, and the "hub neuron" (hn) which is electrically coupled to both of the rhythm-generating parts of the circuit is an intrinsic oscillator of intermediate frequency (plotted dashed lines represent respective intrinsic frequencies)**. The 3 electrically coupled neurons synchronize at a common frequency with sufficient coupling strength when electrical synapses are non-rectifying (case 0) as well as when the polarity and placement of the rectifying electrical synapse cooperates with the intrinsic neuron properties (cases 1 and 4). When the polarity and placement of the rectifying electrical synapse antagonizes the neuron intrinsic properties, one of the rhythm-generating parts of the circuit is cut off from the rest of the circuit (cases 2 and 3).

We find that circuit output depends on the polarity and placement of the rectifying electrical synapse and the intrinsic properties of the neurons on either side of it (Figure 1). Furthermore, using the parameterscape visualization method [3], we find that rectification can affect the circuit's sensitivity to modulation of synaptic strength - including modulation of chemical synapse strength. This can have a dramatic effect on the functional output of a pattern-generating circuit. For a multi-functional motor network, it is important to be able to switch between stable network patterns by synaptic neuromodulation. However, our results show that some kinds of electrical synapse rectification can switch off multi-functionality even when there are degenerate synaptic pathways. In conclusion, these results demonstrate how a rectifying electrical synapse has the potential to specialize a neuronal circuit for robust output or for flexibility.
